# Estimation of the nature and magnitude of mental distress in the population associated with ultra-processed food consumption

**DOI:** 10.3389/fnut.2025.1562286

**Published:** 2025-11-26

**Authors:** Jerzy Bala, Oleksii Sukhoi, Jennifer Jane Newson, Priscila Pereira Machado, Mark Lawrence, Tara C. Thiagarajan

**Affiliations:** 1 Sapien Labs, Arlington, VA, United States; 2 School of Exercise and Nutrition Sciences, Deakin University, Geelong, VIC, Australia; 3 Institute for Physical Activity and Nutrition, Deakin University, Geelong, VIC, Australia

**Keywords:** mental wellbeing, mental health, ultra-processed food, UPF, burden, population health, global health

## Abstract

**Introduction:**

Convincing evidence supports direct associations between exposure to ultra-processed food (UPF) and risks of depressive and anxiety outcomes. However, the impacts of UPF consumption on broader mental wellbeing and functioning and the aggregate clinical burden of mental distress due to these impacts, are currently unknown. This study probes the relationship between various facets of mental wellbeing and UPF consumption and estimates the magnitude of contribution of UPF to adverse mental wellbeing outcomes.

**Methods:**

This cross-sectional study used data from 400,787 respondents across 60 countries in 2023 who completed a comprehensive assessment of mental functioning, together with a broad range of life context factors including UPF consumption frequency. The relationship between mental wellbeing and UPF consumption frequency was calculated controlling for exercise, traumas & adversities and income. Simulations based on tree-based models (XGboost) to capture nonlinearities and cross-level interactions among 108 factors across 10 categories of life context factors along with SHapley Additive exPlanations (SHAP) values were used to estimate the contribution of UPF consumption to mental wellbeing outcomes.

**Results:**

Altogether, there was a systematic decrease in an aggregate metric of mental wellbeing with increased frequency of UPF consumption (*p* < 0.001), contributed by increased symptoms of depression as well as challenges with emotional and cognitive control, even when considering major confounds of income, exercise, life adversity and trauma. Simulations based on predictive models that considered over 10 categories of life context factors estimated that 3.4–7.8% of the global sample experienced clinical mental distress linked to UPF consumption, corresponding to a global UPF-associated clinical mental distress burden of 15.3–28.2%, depending on demographic group, with the burden in the United States and Core Anglosphere higher than the global average.

**Discussion:**

This study provides the first quantitative estimate of the aggregate burden of adverse mental functioning associated with increasingly frequent UPF consumption, calling for greater attention to UPF research and policy as a means to mitigate the mental health burden and strengthening the case for incorporating UPF reduction recommendations into national dietary guidelines.

## Introduction

Ultra-processed foods (UPFs) are generally defined as ‘formulations of ingredients, mostly of exclusive industrial use, that result from a series of industrial processes’ (1) and include fast food dishes, soft drinks, ready-to-heat meals, salty snacks, sugared breakfast cereals, and confectionery. Over the past few decades there has been a global proliferation in their manufacture and consumption, with recent estimates suggesting at least 50% of total dietary energy intake in many high-income countries, and 15–30% in many middle-income countries, comes from UPFs (2–6). UPF consumption, in replacement of whole foods, begins early in life and raises serious concerns for global health (2, 7). Meta-analyses of large-scale population studies indicate a direct association between UPF consumption and over 30 health outcomes, including cardiometabolic risk factors and cardiovascular diseases, gastrointestinal and respiratory conditions, cancer, mental illness and mortality (8–11).

Direct associations between greater UPF exposure and higher risks of depressive and anxiety outcomes are supported by convincing evidence (8, 12–16). However, the magnitude of clinical mental distress (i.e., mental distress that would be of clinical concern) associated with UPF consumption is still unknown, given comorbidity of disorders and symptom profiles that typically do not fit specific disorder categories (17, 18). Furthermore, the World Health Organization (WHO) defines mental health as ‘a state of wellbeing in which the individual realizes his or her own abilities, can cope with the normal stresses of life, can work productively and fruitfully, and is able to make a contribution to his or her community’ (19). With the societal implications of diminished mental health aligned with this definition, there is also a need to consider the impact of UPF consumption on mental wellbeing beyond the presence or absence of psychological distress or clinical symptoms (20, 21).

Here we use cross-sectional data from the Global Mind Project (N = 400,787) to determine the association between frequency of UPF consumption and mental wellbeing evaluated on a sliding scale from distressed to thriving, and estimate the magnitude of this association after controlling for numerous other life context factors. The Global Mind Project uses a comprehensive assessment of mental wellbeing as well as a wide breadth of lifestyle and life experience factors including frequency of UPF consumption, exercise, social relationships, substance use and the experience of adversities and traumas. While cross-sectional data cannot always provide a definitive view of causality (22), such large scale and multi-variate data provides a unique opportunity to parse the magnitude of mental distress associated with UPF consumption after controlling for numerous other factors.

## Methods

### Data source

Data were from the Global Mind Project, a dynamic, ongoing repository of global mental wellbeing and life context data acquired through an online assessment called the Mind Health Quotient (MHQ) assessment (23, 24). Participants were recruited through advertisements placed on Meta and Google that systematically targeted adult age-sex groups within countries and regions of interest across broad based interests and key words. In addition, advertisements were continually and dynamically managed (using Google and Meta Analytics) in response to feedback on the demographic composition of respondents to further ensure sufficient representation across age and sex groups. While this is a non-probability sample, data in the United States (US) sample closely aligns with demographic trends within age-sex groups in the large-scale national American Community Survey (ACS) conducted by the US Census Bureau indicating that the data is broadly representative of the population (25). Alignment for other countries is currently unknown although availability of extensive demographic information allows post-stratification weighting. However, in countries where internet penetration is low, it can only reflect the internet-enabled populations even with post-stratification weighting and therefore must be considered as such (see limitations).

### Data sample

The sample population included 518,064 respondents, aged 18+, across 71 countries who completed the MHQ assessment in 2023 (see Supplementary Table 1 for sample sizes by age, biological sex, country, region and language). Records were removed if time to completion was <7 min; if the same option was selected for all rating questions (standard deviation of answers <0.2); if they responded ‘No’ to the question ‘Did you find this assessment easy to understand?’; and for countries with less than 1,000 responses (see Supplementary Figure 1). 400,787 records across 60 countries were included in the final analysis.

All procedures involving human subjects were approved by the Health Media Lab Institutional Review Board (HML IRB; OHRP Institutional Review Board #00001211, Federal Wide Assurance #00001102, IORG #0000850). Participants took part in the online survey voluntarily, anonymously, and without any financial compensation. Participants consented to take part by clicking on a start button after reading a detailed privacy policy.

Missing data were effectively eliminated by design. Completion of all items was required for submission in order to generate a personalized report, and contextual variables (e.g., UPF, exercise, trauma/adversity) were collected using forced-choice or checklist formats.

### Data elements in the MHQ

The MHQ assessment includes ratings of 47 questions that span symptoms and functions across 10 major mental health disorders as well as elements from Research Domain Criteria (RDoC) (20). It also queries numerous life context factors including demographics, lifestyle behaviors, social relationships, inter-personal trauma experiences, life adversities, medical conditions, substance use, and diet (23, 26) (see Supplementary Table 2 for a full list).

### The MHQ rating scale and score

The 47 mental health questions of the MHQ assessment use a 9-point Likert scale to rate the impact of the item on the ability to function (see Supplementary materials) (23). Ratings from these 47 items were aggregated into a score (the Mind Health Quotient, MHQ) that positioned individuals on a scale from −100 to +200 representing a spectrum from Distressed to Thriving (27). Negative MHQs, on average, equate to 5+ significant symptoms (26), which is typical of a disorder diagnosis, but in this case are disorder agnostic as the MHQ assessment spans symptoms across 10 disorders. MHQ values have been shown to have strong sample-to-sample consistency and criterion validity and relate linearly to the number of self-reported productive days in a month (26, 27). The MHQ assessment has a Cronbach’s alpha of >0.8 for all languages indicating that the 47 elements which form part of the MHQ score represent a consistent construct of ‘mental wellbeing’ and are not fully independent (see Supplementary materials for further information).

### Questions on frequency of UPF consumption and other life context factors

Data on frequency of UPF consumption were obtained using the following question: ‘How often do you eat processed, packaged, or fast food that is not made from fresh ingredients? e.g., McDonalds, Dominos, microwave meals, processed canned food, deli meats/cold cuts, noodles in a cup, packaged crisps/chips, sweets/candies, sodas/fizzy drinks’ with answer options of ‘Several times a day’, ‘Once a day’, ‘A few times a week’; ‘A few times a month’; and ‘Rarely/never’. Questions on exercise, adversities and traumas, and annual household income (see Supplementary materials for answer options) were specifically controlled for, while other demographic factors, social behavior, substance use and medical conditions, were utilized in the models.

### Descriptive analysis

Global MHQ values and the percentage with an MHQ < 0 (% Distressed/Struggling) overall, and for each level of UPF consumption, were obtained by a two-step post-stratification weighting. First, country estimates were obtained by weighting values (e.g., MHQ, UPF frequency, item ratings) by age-sex groups to match national demographics within each country. Validation against Core Anglosphere benchmark data confirmed that this procedure yields distributions closely aligned with national surveys and suggest that values are aligned with the overall population in high internet penetration countries. Second, global estimates were obtained by weighting each country by its internet-enabled population to prevent overrepresentation of high-response countries. Only countries for which >1,000 records were available were used in the analysis (*N* = 60 countries). Where data was insufficient in all age-sex-UPF consumption groups, regional data was grouped (e.g., former Soviet Republics). The Core Anglosphere included the US, Canada, the United Kingdom, Ireland, Australia, and New Zealand.

Descriptive comparisons and t-tests were conducted to visualize the broad dose–response relationship between UPF frequency and MHQ scores, with inferential conclusions drawn instead from multivariate, non-parametric analyses (see *Supervised learning model*). UPF frequency was modeled categorically within a tree-based framework, ensuring that observed associations were not dependent on linear or proportional assumptions (see *Statistical estimation of the impacts of UPF consumption*).

### Statistical estimation of the impacts of UPF consumption

Statistical differences in MHQ and individual rated items between different UPF consumption frequency groups were calculated using a 2-sided t-test for each age-sex-country group. Given that the 47 elements of the MHQ are related to a common underlying construct and not independent, and Type I error corrections (e.g., Bonferroni) apply to independent elements, no correction was applied. The progressive trend in declining MHQ with increased UPF consumption frequency was characterized using linear regression. This was done by computing the significance of the decreasing trend obtained by converting UPF consumption frequency categories into numerical categories as follows: Rarely/Never = 1; A few times a month = 2; A few times a week = 3; Once a day = 4; Several times a day = 5.

### Controlling for confounds of exercise, trauma and adversity, and income

To determine if mental wellbeing trends by UPF consumption frequency were due to secondary effects of other factors, the global trend was compared between groupings of high and low levels of each of exercise, traumas & adversities, and income, individually and all together. The impact of income was only computed for US, India, and Brazil where data on annual household income was available (see limitations). Statistical differences between groups were computed using a standard two tailed t-test.

### Supervised learning model

Numerous other factors beyond those described above (such as social relationships, substance use, education, urbanicity and sleep quality) could influence the estimation of the impact of UPF consumption frequency on mental wellbeing outcomes. As it is difficult to control for each individually, and since many factors are inter-dependent and potentially nonlinear in their effects, we used a tree-based model across all factors to determine their relative impact on the classification of mental wellbeing status. To do this we first employed a one-hot encoding method where items in multiple-choice lists (e.g., different types of trauma experience) were each considered as individual factors coded as 1 (if selected) or 0 (if not selected) resulting in a factor set of 108 features (including age, biological sex, educational attainment, employment status, lifestyle habits including sleep, socializing, exercise, substance use, and various life adversities/traumas; see Supplementary Table 2 for the full list). Next, we used gradient-boosting (XGBoost), which automatically captures nonlinearities and cross-level interactions, to construct a model to distinguish those with mental distress that would be of clinical concern (a negative MHQ reflecting, on average, five or more mental health symptoms (26); hereafter called clinical mental distress) versus those with normal mental wellbeing status (a positive MHQ) using all 108 features including UPF consumption frequency. By including country, age, and sex as predictors, the model effectively accounts for a hierarchical structure without explicit random-effects terms and has been previously shown to perform well with Global Mind data from 2022 that did not include data on UPF consumption (28). For comparison, we also evaluated model results using Logistic Regression, Naïve Bayes and Random Forest.

Three-, five-, and ten-fold cross-validation was performed with five evaluation metrics: area under the ROC curve (AUC), classification accuracy (CA), precision, recall and F1 score (the harmonic mean of precision and recall), to evaluate and compare the algorithms. Cross-validation results for each metric were averaged across folds to obtain intermediate benchmark performance estimates. Final reported results were obtained using a 65/45 train/test split, randomly generated five times, to evaluate performance on unseen (test) data. Results are reported as average performance across positive and negative MHQ prediction models over the five test sets (see Supplementary materials for more information).

### Assessing the impact of life context factors on MHQ predictions

To assess the impact of specific life context factors on prediction outcomes in this XGboost classifier model, we computed SHAP (SHapley Additive exPlanations) values. Positive SHAP values reflected instances that moved the classification toward a positive MHQ while negative SHAP values reflected instances that moved the classification toward a negative MHQ. These values were then visualized in a bee swarm plot.

### Estimation of the magnitude of mental distress in the sample population associated with UPF consumption

In order to control for all 108 features measured in the study, we estimated the magnitude of clinical mental distress in the sample population associated with UPF consumption by simulating a change in UPF consumption using the XGboost classification model described above. Here, we set the UPF consumption frequency for all records in the test to ‘Rarely/Never’ without changing any other features and re-ran the model. We then computed the difference between the percentage predicted to have a negative MHQ using the model before and after the manipulation, for broad age-sex groups for US, the Core Anglosphere and for global data. This value represents the percentage of the sample estimated to have mental distress linked to UPF consumption. The estimated reduction in clinical mental distress associated with eliminating UPF consumption (hereafter called UPF-associated clinical mental distress burden) was then calculated by dividing this by the percentage of individuals classified as Distressed/Struggling in the sample prior to any change in the UPF consumption frequency distribution. We repeated this with 10 iterations of randomly selected test data and report the average values across these iterations.

## Results

These results represent responses of 400,787 male (46%) and female (54%) respondents aged 18+, spanning 60 countries who completed the MHQ in 2023.

### Overall mental wellbeing decreases with frequent UPF consumption

**Figure 1:**
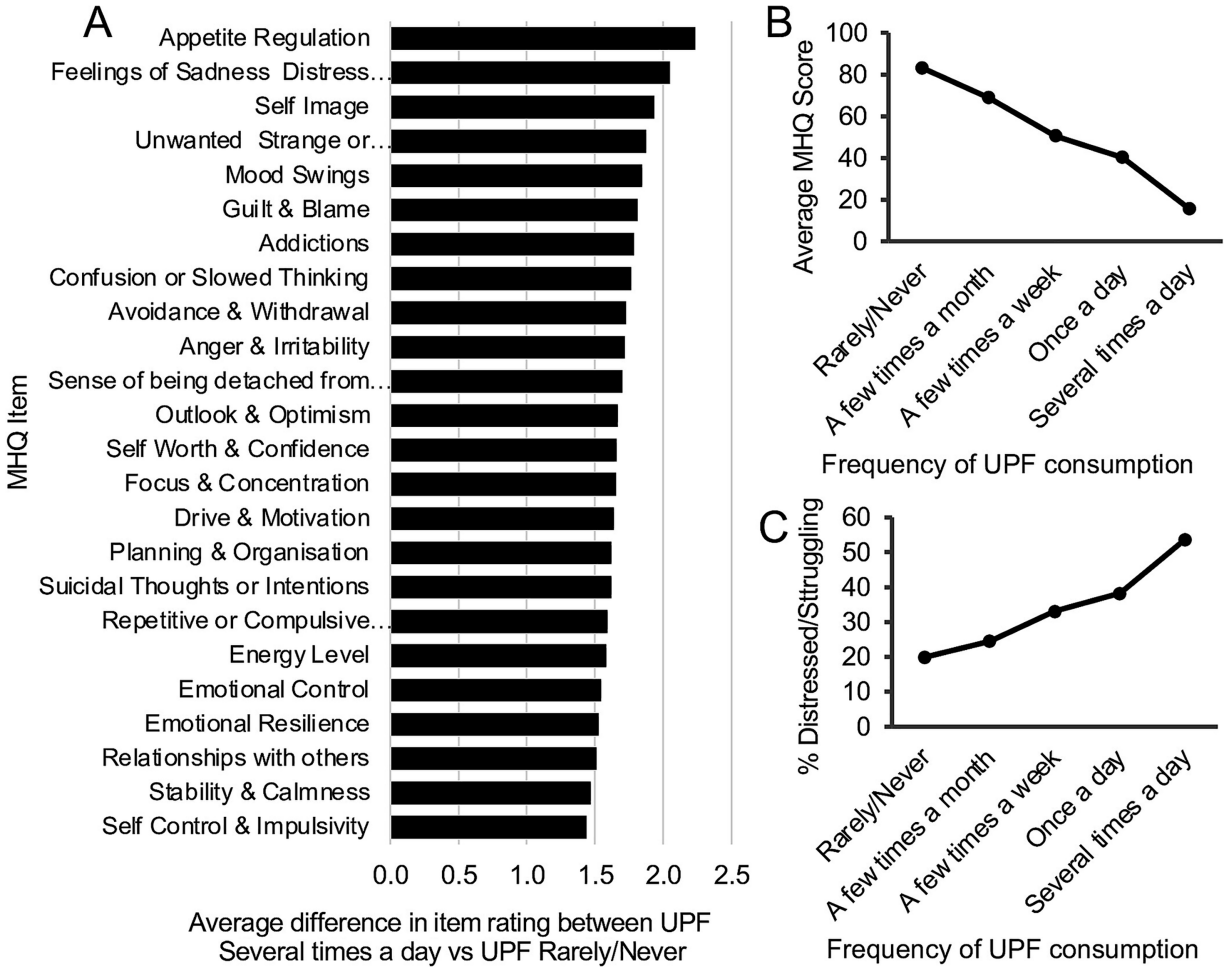
Mental wellbeing and UPF consumption frequency. **(A)** Difference in average life impact ratings between those who consumed UPF ‘Several times a day’ compared to those who consumed it ‘Rarely/Never’, for items with difference greater than ±1.5 on the 9-point scale (difference of ≤ − 1.5 for spectrum items and ≥ + 1.5 for problem items, indicating a worse rating). All *p* < 0.001. **(B)** Relationship between average MHQ and frequency of UPF consumption (mean ±SEM). Note the MHQ ranges from −200 to +100 and only the relevant score range has been shown. **(C)** Relationship between percentage Distressed/Struggling (MHQ < 0) and frequency of UPF consumption.

Among this global internet-enabled population sample, average mental wellbeing, measured by the MHQ, was 63.4 in the range considered ‘Managing’, with 27.7% having a negative MHQ or clinical mental distress. 31.1% indicated they consumed UPF at least once a week (an aggregate of ‘Several times a day’, ‘Once a day’, ‘A few times a week’) while 38.1% consumed UPF ‘Rarely/Never’. Altogether, ratings for all 47 items decreased significantly with increasing frequency of UPF consumption, suggesting a broad association between UPF consumption frequency and social, emotional, and cognitive functioning (Figure 1A; Supplementary Table 3). The top 3 items, as well as 5 out of the top 10 items in Figure 1A, were key symptoms associated with depression, while the other items included challenges to the control of emotions and thoughts. The trend was similar across all countries with only small differences in the relative impact of items (see Supplementary Table 4).

The aggregate mental wellbeing score, the MHQ, computed from these 47 items decreased systematically and significantly (*p* < 0.001) with each higher frequency of UPF consumption, with a difference of 67.6 MHQ points between the highest and lowest frequency groups, representing 22.5% of the 300-point MHQ scale (global data in Figure 1B, individual countries and trend statistics in Supplementary Table 5). Correspondingly, the percentage with clinical mental distress (i.e., MHQ < 0) increased 3-fold (19.9% vs. 53.7%) between those who ‘Rarely/Never’ consumed UPF, and ‘Several times a day’ (Figure 1C; Supplementary Table 5). Moreover, with 27.7% of the sample experiencing clinical mental distress (across all UPF consumption frequencies; not shown here) compared to 19.9% among those who ‘Rarely/Never’ consumed UPF, this suggests that up to 7.8% (27.7–19.9%) of this global sample could be experiencing mental distress linked to UPF consumption corresponding to a UPF-associated clinical mental distress burden of 28.2% (7.8%/27.7%). In the internet-enabled US, Brazil and India, the difference in the percentage experiencing mental distress between those who ‘Rarely/Never’ consumed UPF, and ‘Several times a day’ was 30.1, 34.5 and 40.6%, respectively (Supplementary Table 5), while prevalence of UPF consumption at least once a week or more was 55.3, 25.4 and 28.8%, respectively.

### Ruling out major confounds of exercise, trauma and adversity, and income

**Figure 2:**
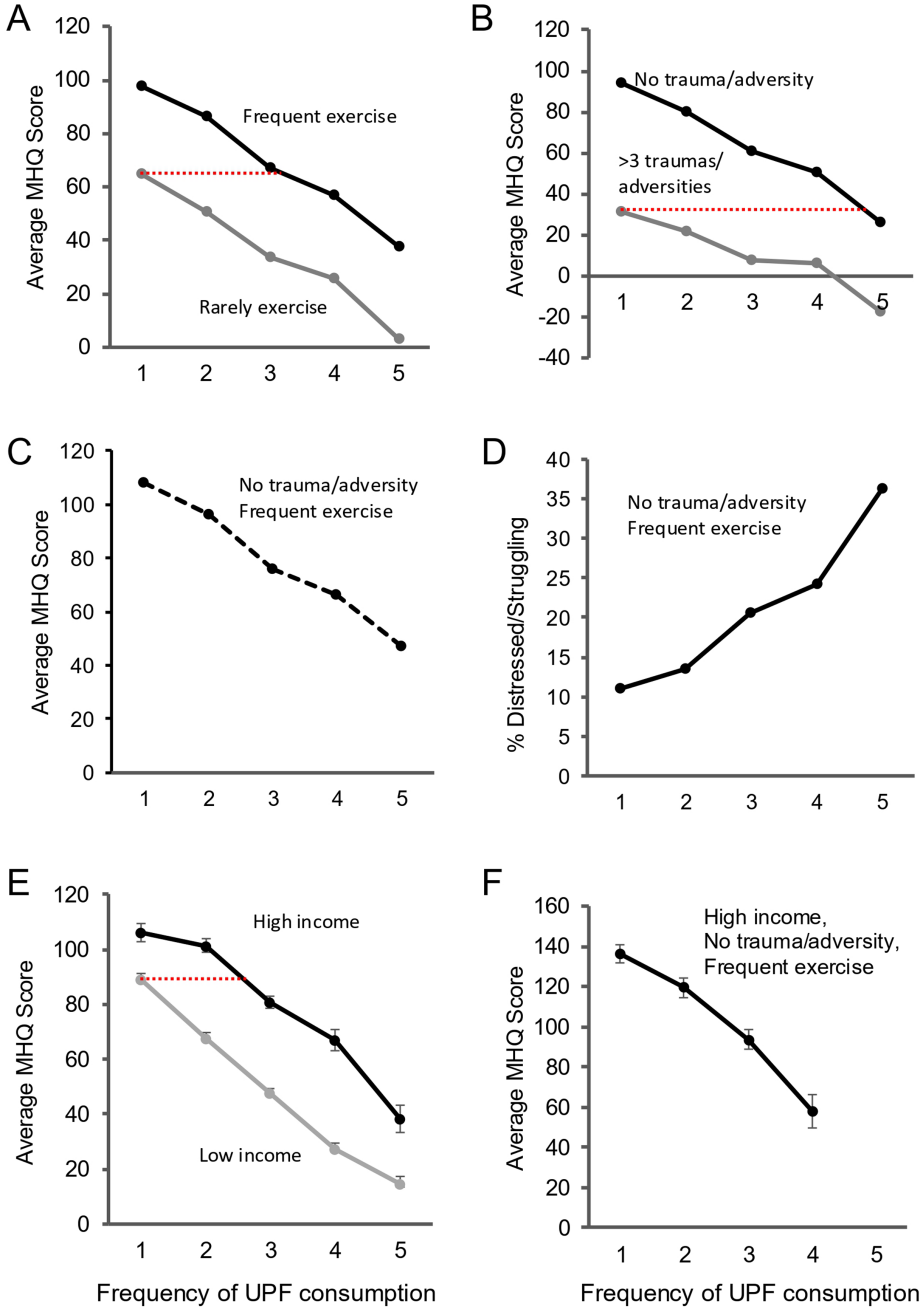
The impact of UPF consumption frequency persists after controlling for exercise, traumas/adversities and income individually and together. Frequency UPF Consumption: 1 = Rarely/Never; 2 = A few times a month; 3 = A few times a week; 4 = Once a day; 5 = Several times a day. Note the MHQ ranges from −200 to +100 and only the relevant score range has been shown. **(A)** Average MHQ (global sample) across different levels of UPF consumption frequency for frequent exercise (Everyday+Several times a week; black) and infrequent exercise (Less than once a week+Rarely/Never; gray). **(B)** Average MHQ (global sample) across different levels of UPF consumption frequency for no listed traumas/adversities (black) and ≥3 listed traumas/adversities (gray). **(C)** Average MHQ (global sample) across different levels of UPF consumption frequency for no trauma/frequent exercise combined. **(D)** Percentage Distressed/Struggling (global sample) across different levels of UPF consumption frequency for no trauma/frequent exercise combined. Note the different y axis. **(E)** Average MHQ (US only) across different levels of UPF consumption frequency for high income (black) and low income (gray). **(F)** Average MHQ (US only) across different levels of UPF consumption frequency for high income/no trauma/frequent exercise combined. Dotted red lines indicate the MHQ in the higher exercise/education/income groups equivalent to that of the lowest UPF consumption frequency in the lower groups.

Lower income, lack of exercise and experience of traumas and adversities have all been associated with greater UPF consumption (29–34) and impact various aspects of mental health (35–37). In this data, UPF consumption was more frequent in lower income groups in developed countries (but the opposite in developing countries such as India), and also among those who were sedentary or had experienced greater adversity and trauma (Supplementary Table 6). Here we show that controlling for exercise, traumas and adversities, and income each individually, and all together, did not substantially diminish the association between UPF consumption frequency and mental wellbeing. Figure 2A shows that the decline in MHQ with increasing UPF consumption frequency in the global data for those who exercised several times a week or more (60.5 MHQ points between UPF consumption of ‘Rarely/never’ and ‘Several times a day’) was similar to those who exercised less than once a week (61.9 MHQ points). In addition, the difference between the two lines (31–35 MHQ points across all frequencies of UPF consumption) suggests that exercise had an independent effect on MHQ. Figure 2B shows a similar trend for those who had experienced ≥3 traumas/adversities (49.1 MHQ points between UPF consumption of ‘Rarely/never’ and ‘Several times a day’) and those who had experienced no traumas/adversities (68.1 MHQ points). Figure 2C shows the decline in MHQ with increasing UPF consumption frequency controlling for both together (60.5 MHQ points between UPF consumption of ‘Rarely/never’ and ‘Several times a day’). Correspondingly, Figure 2D shows a 25.3% increase in the percentage Distressed/Struggling between UPF consumption of ‘Rarely/never’ and ‘Several times a day’ globally for those who exercised frequently and had experienced no traumas/adversities. A similar pattern of results was obtained for US, India and Brazil (Supplementary Table 7).

Figure 2E shows a similar change in MHQ with increasing UPF consumption for those with annual household income ≤$40,000 (74.3 MHQ points between UPF consumption of ‘Rarely/never’ and ‘Several times a day’) compared to those with an annual household income ≥$100,000 in the US (67.8 MHQ points; other countries in Supplementary Table 7). Significantly, while income had an independent effect (difference between lines; 17–40 MHQ points depending on UPF consumption frequency), someone with an annual household income of ≥$100,000 who consumed UPF at least once a week had, on average, a similar MHQ to someone with an annual household income ≤$40,000 who ‘Rarely/Never’ consumed UPF (red line, Figure 2E). Here again, controlling for income, exercise and adversities/ traumas together did not substantially diminish the impact of UPF consumption on mental wellbeing (57.1 MHQ points; Figure 2F) or the percentage Distressed/Struggling (difference 9.0%).

### A model approach to estimating the impact of UPF consumption

The descriptive comparisons and *t*-tests above were conducted to visualize the broad directional relationship between UPF consumption frequency and MHQ scores and difference between both extremes. However, as the categories are ordinal and as relationships are unlikely to be linear, we next looked at multivariate, non-parametric approaches. To do so, UPF frequency was modeled categorically within a tree-based framework which ensures that observed associations were not dependent on linear or proportional assumptions. This also allows for the inclusion of numerous other factors that could also impact mental wellbeing in confounding ways. We specifically used the tree-based XGBoost classification model to determine the association between UPF consumption and mental wellbeing status when considering all other factors together. Overall, the model was able to classify individuals with accuracy of 0.82, precision of 0.81 and recall of 0.82. Performance of other model types Logistic Regression, Naïve Bayes, and Random Forest was similar (AUC = 0.82–0.85; accuracy = 0.77–0.81; F1 = 0.79–0.81; Supplementary Table 8), demonstrating that the UPF–wellbeing association is robust across diverse modeling frameworks.

**Figure 3:**
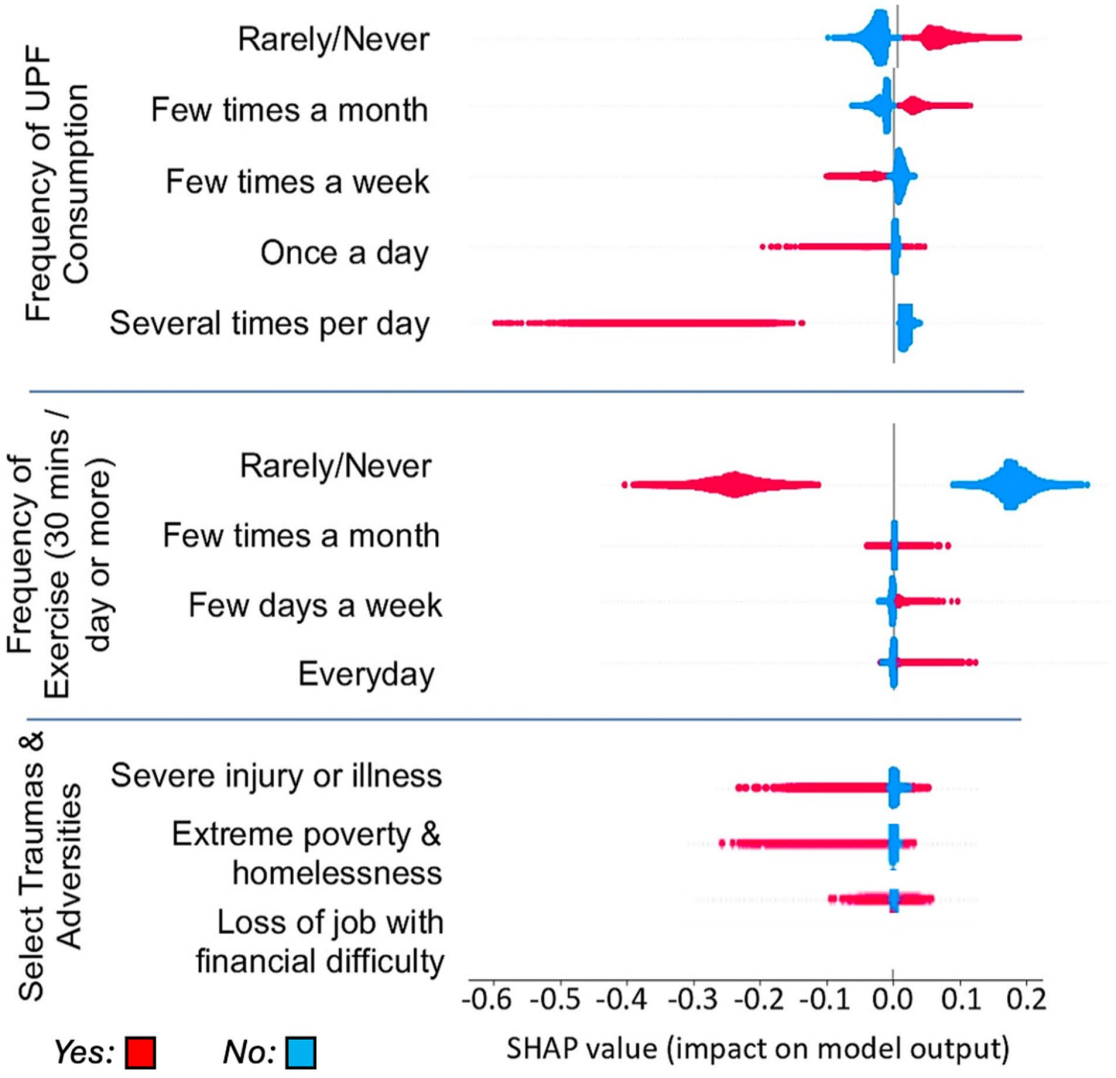
SHAP values for each of the UPF consumption frequency answers in comparison to exercise frequency answers and answers for select individual adversities & traumas.

Given the high degree of correlation among factors, removing UPF consumption frequency as a variable from the data resulted in only a small change in model performance. As XGBoost has the advantage of providing interpretable SHAP-based feature attributions we examined the specific impact of UPF consumption frequency within the model using SHAP outcomes. Figure 3 shows the SHAP outcomes for several relevant factors in the model including each UPF frequency, exercise frequency and examples of adversities including injury or illness, homelessness, or loss of a job. Each individual is represented by a dot where red dots indicate those who selected that option, and blue those who did not select that option. Their position along the x axis indicates how much that particular selection shifted the classification toward a negative or positive MHQ. The impact value assigned for each individual varies based on which tree is used, taking into consideration all factors within the tree. While UPF frequency selections of ‘Rarely/never’ and ‘A few times a month’ resulted in a shift toward a positive MHQ classification, ‘A few times a week’ to ‘Several times a day’ resulted in increasingly negative MHQ. Similarly, a selection of ‘Rarely/never’ for exercise resulted in a negative impact on MHQ while increasing exercise frequency had positive impacts. In addition, the experience of a severe illness or injury, and extreme poverty leading to homelessness during childhood or adulthood, had negative impacts, while loss of a job leading to financial difficulty had a mixed impact. Overall, UPF consumption frequency of ‘Several times a day’ had the most negative impact on MHQ of all these factors, although we note this ordering varied across individuals.

### Model validation and sensitivity analyses

To assess model reliability, we performed additional experiments evaluating (i) SHAP feature-importance stability across 5/10 cross-validation folds, (ii) sensitivity to correlated predictors, and (iii) the independent contribution of UPF frequency. SHAP feature rankings were highly consistent (mean Spearman *ρ* ≈ 0.93), computed across all pairwise fold comparisons, confirming stable feature ranking. Removing features with pairwise |r| > 0.80 had a negligible effect on model performance (ΔAUC ≈ 0.01), indicating robustness to multicollinearity. Excluding all UPF-related variables reduced AUC by approximately 0.03, demonstrating that UPF consumption frequency contributes unique predictive information beyond other contextual factors. Calibration curves (Supplementary Figure 2) further confirmed appropriate probability behavior. These analyses collectively confirm the stability and interpretability of SHAP contributions, supporting the reliability of the XGBoost-based modeling framework.

### Estimating the magnitude of clinical mental distress associated with UPF consumption

**Figure 4:**
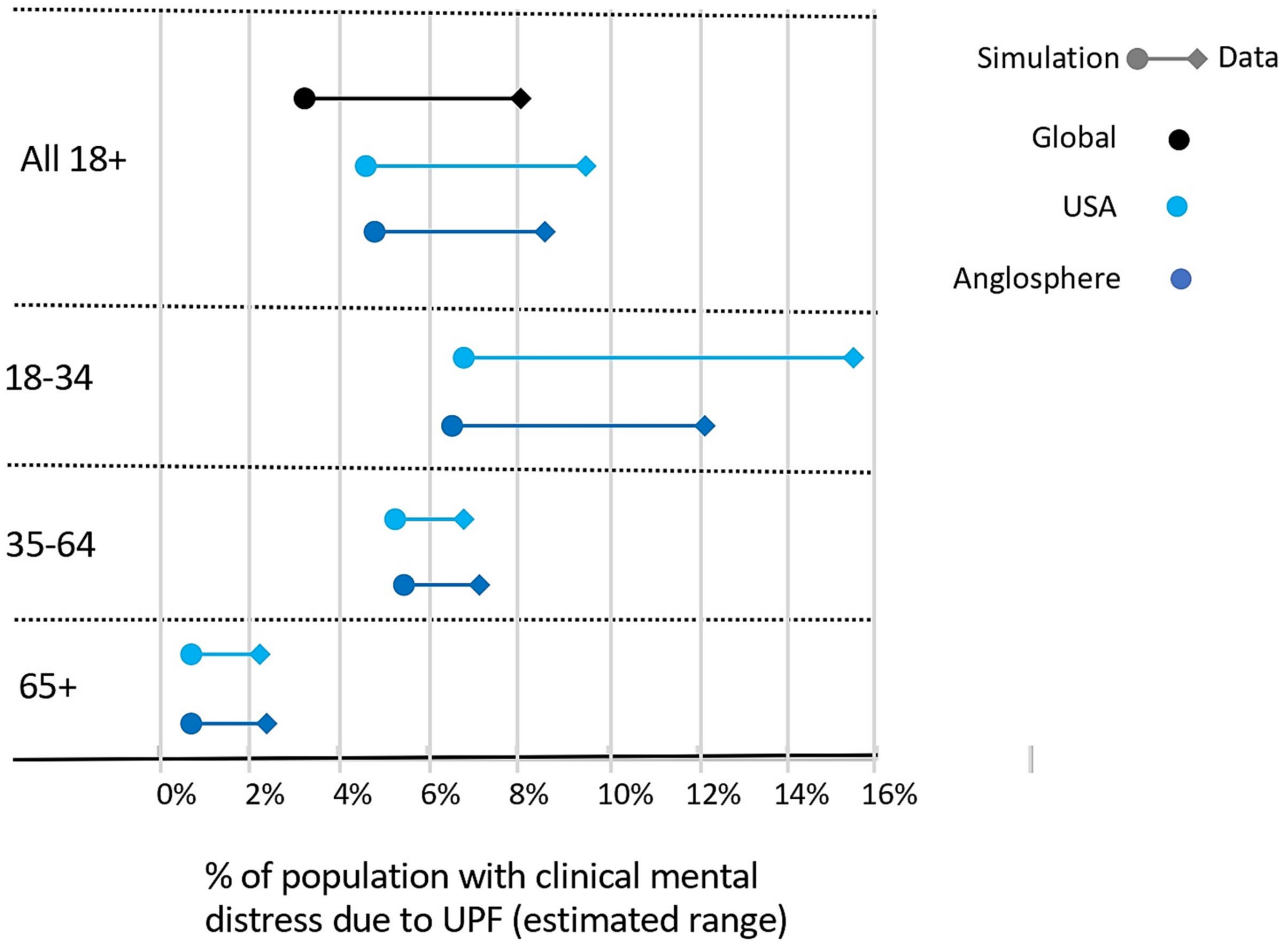
Estimated percentage of sample with UPF-associated clinical mental distress across age groups using a model-based simulation that considers a large range of other lifestyle and life experience factors including substance use, medical conditions and social factors.

Without controlling for other factors, the analysis above suggested that up to 7.8% of the global sample could be experiencing clinical mental distress linked to UPF consumption, corresponding to a UPF-associated clinical mental distress burden of 28.2%. Here we used a simulation-based approach applied to the XGBoost classification model to determine the magnitude of mental distress associated with UPF consumption when taking into consideration all 108 life context features including age, biological sex, educational attainment, employment status, substance use, lifestyle, life trauma/adversity. Using this simulation method, 3.4% of the global sample were estimated to have clinical mental distress linked to UPF consumption corresponding to a UPF-associated clinical mental distress burden of 15.3%. Figure 4 shows the range between the data and simulation-based estimates.

However, accurate global estimates are challenging due to insufficient data in all age-sex-UPF consumption groups for certain countries and because the samples in developing countries where internet penetration is low are not representative. Therefore, for estimates by age we looked specifically at the US where the sample has been shown to be broadly representative of the population (25), and Core Anglosphere where Internet penetration is high, and sample sizes are larger and therefore more likely to be representative (Figure 4; Table 1; Supplementary Table 9). Overall, the estimated percentage of the sample experiencing clinical mental distress linked to UPF consumption was highest for males aged 18–34 in the US (7.0% simulation, 17.4% data). For older adults (65+), where frequency of UPF consumption was universally low, estimates were lower with no substantial difference between the US (0.7% simulation, 2.3% data) and Core Anglosphere (0.9% simulation, 2.4% data), and were lowest for females. In addition, although estimates were substantially lower in in the 65 + group, as the overall percentage of individuals with MHQ < 0 was also lower in this group these numbers represented a proportionally higher fraction. Altogether, across both methods, in the US and Core Anglosphere, 5 to 9 out of every 100 adults (aged 18+) and 7 to 14 out of every 100 young adults (aged 18–34) were estimated to have clinical mental distress linked to UPF consumption, corresponding to a UPF-associated clinical mental distress burden of 17 to 32% (aged 18+) and 16 to 35% (aged 18–34). Given lower prevalence of UPF consumption in age groups above 25 in developing countries, global estimates are likely to be lower.

**Table 1:** Estimates of clinical mental distress burden associated with UPF consumption using model-based simulation for the global, US and Core Anglosphere samples.

	Approach	% of the sample experiencing clinical mental distress linked to UPF consumption			% reduction in clinical mental distress associated with eliminating UPF consumption (UPF-associated clinical mental distress burden)		
Age		Global	US	Anglosphere	Global	US	Anglosphere
All	Data	7.8%	9.4%	8.5%	28.2%	31.9%	28.0%
	Simulation	3.4%	4.7%	4.8%	15.3%	18.7%	17.2%
18–34	Data		15.5%	12.1%		35.0%	27.0%
	Simulation		6.7%	6.7%		17.3%	15.8%
35–64	Data		6.7%	7.0%		28.0%	27.0%
	Simulation		5.2%	5.3%		18.0%	16.4%
65+	Data		2.3%	2.4%		29.0%	29.0%
	Simulation		0.7%	0.9%		23.0%	21.8%

## Discussion

To date, studies examining the impact of UPF consumption on mental health have typically focused on symptoms associated with depression or anxiety (13, 38–45). Here we have shown (1) that the impact of UPF extends beyond symptoms of depression and anxiety and includes a broad range of mental functioning (2), that the impact of UPF cannot be attributed to secondary effects of income, exercise, or adversities and trauma, and (3) that the magnitude of clinical mental distress associated with UPF consumption may be in the range of 15 and 28%, with differences depending on age and geography.

### Frequent UPF consumption has a broad impact on mental functioning

A higher frequency of UPF consumption was systematically associated with more negative ratings on all 47 MHQ items assessed, which include symptoms of 10 major mental health disorders. Consistent with numerous other studies demonstrating an association between UPF consumption and depression (8, 12, 16), the items with the largest rating differential included depression-associated symptoms of poorer appetite control, greater feelings of sadness, distress or hopelessness and lower energy levels. However, numerous other symptoms were also associated with UPF consumption including greater challenges with focus & concentration, unwanted thoughts, anger and emotional control. Overall, this translated to a difference of 67.6 MHQ points between people who consumed UPF several times a day, compared to rarely or never, a difference of 22.5% on the MHQ scale. This difference also persisted after controlling for major confounds of income, exercise, and trauma/adversity. Thus, there was a progressive downward shift in mental functioning with more frequent UPF consumption, rather than a sudden shift from the absence of a problem to the presence of a problem. The implication is that more frequent UPF consumption diminishes mental wellbeing and functioning, increasing prevalence of sadness, decreasing energy levels, and leaving people with lower capacity for emotional and cognitive control. The consequence of growing levels of UPF consumption around the world (2–6), therefore extends far beyond a disorder context, and has wider implications for general productivity and functioning of society.

### UPF consumption and exercise

While the association between frequent UPF consumption and poor mental wellbeing persisted after controlling for exercise, those who exercised regularly had substantially higher mental wellbeing at all frequencies of UPF consumption, indicating that the effects of good diet and exercise may be largely additive (and you cannot ‘out run’ a poor diet). The SHAP analysis revealed that while consuming UPF multiple times a day had a larger negative effect than lack of exercise overall, the effect of lack of exercise was more consistent across individuals. This could be due to variable patterns in the types of UPF consumed among respondents.

### The impact of UPF on mental wellbeing in an economic context

Increasing income is often considered the path to greater wellbeing and prosperity. However, this data shows that someone with high income who consumed UPF several times a day had poorer mental wellbeing, on average, compared to someone with low income who rarely or never consumed UPF. Furthermore, the SHAP values show the impact of daily consumption of UPF contributed more to the prediction of poor mental wellbeing than significant financial factors such as poverty and homelessness or loss of a job. Put differently, the findings suggest not only that higher income does not counter the effects of frequent UPF consumption on mental wellbeing, but that spending that income on UPF likely negates its benefits. This provides food for thought in terms of how we might approach the trade-off between UPF regulation and economic growth.

### Estimation of the UPF-associated clinical mental distress burden

In this study we considered clinical mental distress to be a negative MHQ which roughly corresponds to 5 or more clinical level symptoms, similar to the threshold for diagnosis of many mental health disorders as defined by the DSM-5 (26). However, in this case these symptoms are not specific to any one disorder definition due to the transdiagnostic nature of the MHQ assessment. In these terms, we estimated that between 3.4 and 7.8% of this large global sample may be experiencing clinically relevant mental distress associated with frequent UPF consumption, corresponding to a global UPF-associated clinical mental distress burden of 15.3 to 28.2% (where the lower estimates are derived from simulations that control for all other factors and the upper estimates are based on the differences in the data without controlling for other factors). Estimates were also higher for the US and Core Anglosphere, for younger age groups, and in males. The highest estimates were for males aged 18–34 in the US where estimates were 17.4% of the sample, corresponding to a UPF-associated clinical mental distress burden of 43.0%. For those aged 65+, there were minimal geographic differences possibly because these older generations grew up in an era before the excessive proliferation of UPF. The lowest estimates were for 65+ females (1.2–1.7% of the sample). We note, however, that the simulation assumes no indirect effects of UPF consumption on other life context factors such as exercise, social interaction or substance use which could drive secondary impacts on mental wellbeing outcomes. For example, UPFs are often ‘ready-to-eat’ convenience products designed to be consumed anywhere, at any time, affecting the socialization of cooking and mealtime (46) and displacing culturally appropriate dietary patterns (46, 47). Furthermore, while we have controlled for inter-personal traumas and demonstrated that the effects of UPF persist, we cannot rule out that UPF consumption leads to greater interpersonal conflict that would, in turn, increase mental distress. These estimates therefore represent a floor, where, in reality the UPF-associated clinical mental distress burden could be somewhere in between the simulation estimates and the (uncontrolled) estimates from the data. Furthermore, as UPF consumption continues to increase around the world, its societal impact is likely to only grow further.

### Possible mechanisms of UPF impact

UPFs contain a range of chemical additives (e.g., non-nutritive sweeteners, artificial flavors and colors, preservatives, stabilizers, and emulsifiers), as well as contaminants (e.g., advanced glycation end-products) that have been implicated in the modulation of biological pathways relevant to mental disorders. For example, frequent UPF consumption has been shown to disrupt the gut microbiome and, in turn, the gut–brain axis (48, 49). It has also been associated with myelin degradation (50), changes in gray matter volume and inflammatory biomarkers (40), and dysregulation of reward signaling (51). However, further research is needed to disentangle the specific effects of individual classes of chemical ingredients and their cumulative impact on the brain.

In addition, diets high in UPFs are typically characterized by poor nutritional quality, and low levels of essential micronutrients, such as vitamins, minerals, and omega-3 fatty acids, which are critical for brain structure and function (52, 53). The high energy density but low nutrient density of UPFs means they can displace more nutrient-rich whole foods in the diet, leading to micronutrient deficiencies that may compromise neural development and maintenance.

### Policy implications

These results add to the growing body of literature which suggest there is substantial benefit to reducing UPF intake on the global and national mental health burdens (8, 12, 53). Such a preventative approach is further bolstered by studies showing that even 3 weeks of eliminating UPF from the diet can impact depression symptoms (54). This also adds to the case for UPFs to be considered in the preparation of global and national burden of disease reports, as well as to calls for incorporating UPF reduction recommendations into national dietary guidelines (55). A range of policy interventions are available to promote the substitution of minimally processed foods for UPFs such as warning statements on the labels of UPFs, positive statements on labels of minimally processed foods, and using income from a UPF tax to subsidize the cost of minimally processed foods such as fruits and vegetables. In addition, when setting food standards, the scope of risk assessment of industrial food ingredients needs to be extended to include their long-term cumulative impact on chronic disease outcomes, including mental health.

### Strengths and limitations

While cross-sectional studies assess exposure and outcome simultaneously, limiting their ability to establish causality due to challenges in determining timing and potential reverse causation, they can nonetheless provide valuable insights and generate hypotheses about potential causal links (22). Moreover, such cross-sectional studies are both timely and feasible on a large scale, particularly for the purpose of population-level estimation where the cost and logistics of interventional or longitudinal studies would be prohibitive. In this case, the breadth of variables, studied simultaneously across a large global sample, confers the ability to establish causal plausibility by controlling for numerous potential confounds compared to univariate approaches. However, there are limitations to note. Firstly, asking individuals to estimate their frequency of food intake can be impacted by recall bias. Secondly, UPF was measured as a single overarching question, due to the time practicalities of asking individuals to complete an online assessment. Third, despite several UPF examples being provided, it is possible that these examples are not representative of the overall UPF dietary pattern in all countries, which could lead to a potential underestimation. In addition, there are differences in opinion and definition in terms the scope of items considered ‘ultra-processed’. For example, canned foods are not always considered ultra-processed yet typically include preservatives and so were included here. Nevertheless, the items included are among the largest contributors to UPF sales worldwide (2) and therefore account for a substantial proportion of available UPFs. Fourth, not every possible life context factor was included in the MHQ assessment due to constraints of the length of the assessment and ensuring adequate completion rates. In addition, data on household income was only available for 3 countries due to challenges with ensuring equivalence across countries when numerical values are used. Finally, the coverage within the 60 countries only represents internet-enabled populations where UPF consumption may be higher. Thus, global estimates may be less reliable than estimates for the US alone where the data has been shown to align with national statistics.

### Future research directions

Altogether, while not causally conclusive, the magnitude of the UPF-associated mental distress shown here makes a strong case for substantially increasing mental health research funding in this area. Future research priorities that could strengthen the evidence base to inform policy actions that replace UPF intake with minimally processed food are as follows. First, observational studies to examine the association in young children and in an extended range of countries. Second, experimental studies to investigate if reduced UPF dietary intake and/or increased minimally processed food dietary intake can mitigate adverse mental health symptoms and the dose–response nature of the association. Third, controlled trials on specific UPF products out of the range specified, or specific UPF ingredients, to determine which may contribute more to adverse effects. Finally, mechanistic research to investigate UPF’s chemical components and/or physical structures that explain how they affect mental health and the physiological, immunological and/or hormonal pathways through which they act.

## Conclusion

The impact of UPF consumption on mental health, wellbeing and functioning is a new and important area of research. Dietary risk factors are well-established as leading contributors to the global burden of disease through their association with non-communicable diseases (NCDs), obesity and nutritional deficiencies. This study provides valuable evidence for a large adverse impact of frequent UPF consumption on mental functioning that has sweeping societal consequences and implications for both future research, as well as food and nutrition policy.

## Data Availability

The datasets presented in this study can be found in online repositories. The names of the repository/repositories and accession number(s) can be found at: https://sapienlabs.org/global-mind-project/researcher-hub/.
