# Supplementary figures and images for: Estimation of the nature and magnitude of mental distress in the population associated with ultra-processed food consumption
Source: Front Nutr. 2025 Nov 26;12:1562286. doi: 10.3389/fnut.2025.1562286 (PMC12689381; doi:10.3389/fnut.2025.1562286)

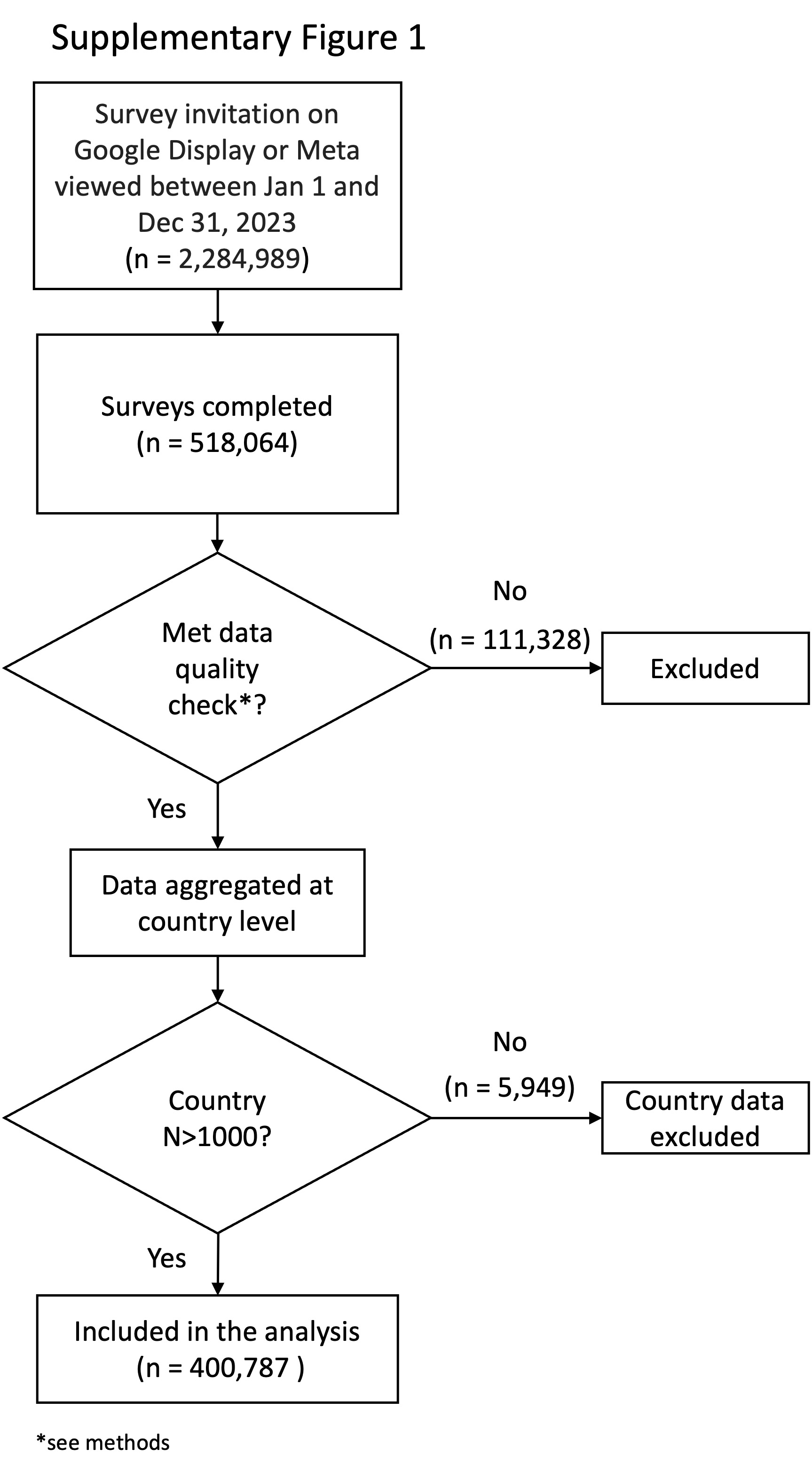

Supplement: Supplementary file 1 [file Image_1.jpeg]

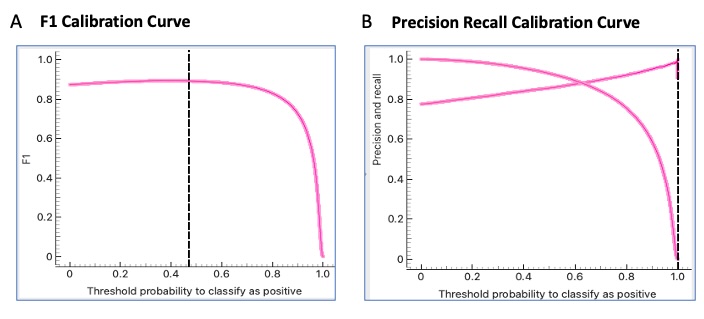

Supplement: Supplementary file 2 [file Image_2.jpeg]
