# Supplementary material for: Estimation of the nature and magnitude of mental distress in the population associated with ultra-processed food consumption
Source: Front Nutr. 2025 Nov 26;12:1562286. doi: 10.3389/fnut.2025.1562286 (PMC12689381; doi:10.3389/fnut.2025.1562286)
Supplement: Supplementary file 4 [file Table_2.docx]

**Estimation of the nature and magnitude of mental distress in the population associated with ultra-processed food (UPF) consumption**

**Supplementary materials**

**The MHQ rating Scale:**

The 47 items of the MHQ assessment were rated by respondents using a 9-point life impact scale (i.e., a Likert scale with 9 positions) reflecting the impact on one's ability to function. For items on a spectrum from positive to negative (spectrum items such as “Memory”) 1 on the 9-point scale referred to “Is a real challenge and impacts my ability to function”, 9 referred to “It is a real asset to my life and my performance” and 5 referred to “Sometimes I wish it was better, but it's ok”.  For items with varying degrees of problem severity (problem items such as “Suicidal thoughts & intentions”) 1 on the 9-point scale referred to “Never causes me any problems”, 9 referred to “Has a constant and severe impact on my ability to function” and 5 referred to “Sometimes causes me difficulties or distress but I can manage”. Respondents made rating responses based on their current perception of themselves.

**The MHQ Score**

Ratings from these 47 items were aggregated into a score (the MHQ score) that positioned individuals on a spectrum from Distressed (-100) to Thriving (+200). The score is based on an algorithm that thresholds ratings as negative and positive based on the impact to function and applies a nonlinear transformation of the scale such that increasing negative impact to function is amplified. The resulting MHQ scores fall on a positive-negative continuum. The positive scores range from 0 to 200 and were scaled to a mean of 100 based on sample data from 2019 (obtained from the United States (US), United Kingdom (UK) and India, English speaking population pre-COVID-19 pandemic). The negative side of the scale has the structure of a long tail that has been linearly rescaled to compress values within a range of -1 to -100. The MHQ score has been shown to have strong sample-to-sample consistency as well as criterion validity using data from 179,298 people across eight English-speaking countries. This includes demonstration that, in the aggregate, average number of clinical symptoms and clinical diagnoses increase systematically as MHQ scores decrease, and that MHQ scores are linearly related to work productivity, including absenteeism and presenteeism.

**Answer options for exercise and annual household income questions**

Specifically, data on physical exercise was obtained with the following question: “How regularly do you engage in physical exercise (30 minutes or more)?” with answer options of ‘Every Day’; ‘Few days a week’; ‘Once a week’; ‘Less than once a week’; ‘Rarely/Never’. Annual household income data was obtained with the following question: “What is your annual household income? (the sum of income from all sources received by all members of the household who operate as a financial unit in a year)”. Income was available for select countries only (US, India, Brazil). For the US, household income answer options were ‘Under $20,000’; ‘$20,001 – $40,000’; ‘$40,001 – $60,000’; ‘$60,001 – $80,000’; ‘$80,001 – $100,000’; ‘$100,001 -$250,000’; “More than $250,000’. For India, answer options were “Less than INR 1,00,000’; ‘INR 1,00,001 – 3,00,000”; “INR 3,00,001 – 5,00,000’; ‘INR 5,00,001 – 10,00,000’; ‘INR 10,00,001 – 20,00,000’; ‘INR 20,00,001 – 40,00,000’; ‘More than INR 40,00,000’.  For Brazil, answer options were: ‘Até 3 salários mínimos’ (i.e., salary up to 3 monthly minimum wage); ‘3-10 salários mínimos’; ‘10-20 salários mínimos’; ‘20-30 salários mínimos’; ‘Mais de 30 salários mínimos’.  Data on traumas and adversities were obtained by the question ‘Have you experienced any of the following during your adult life (since age 18)?’ (see Supplementary Table 2 for a list of traumas and adversities).

**Controlling for major confounds of exercise, adversity and income**

For the global data this included a comparison of the change in mental wellbeing with UPF consumption frequency 1) between those who exercised frequently (‘Everyday’ or ‘Several times a week’) versus infrequently (‘Less than once a week’ or ‘Rarely/Never’); 2) between those who indicated they had experienced none of a set of listed adversities or traumas in their adult life (no listed traumas/adversities) versus three or more (≥3 listed traumas/adversities); 3) for the combinations of both these together – i.e. those who exercised frequently and had experienced no listed traumas/adversities. As data on annual household income was only available for a subset of countries (US, India and Brazil), to examine the impact of annual household income, both alone and in combination with exercise and life adversity/ trauma, data from these 3 countries were examined individually. To examine the impact of annual household income, both alone and in combination with exercise and life adversity/ trauma, we conducted the same comparisons as for the global, as well as 1) the comparison between low income [≤$40,000 (US); ≤ 3,00,000 INR (India); ≤ 3 salários mínimos” (Brazil)] and high income [≥$100,000 (US); ≥ 10,00,001 INR (India); ≥ 10 salários mínimos (Brazil)]; 2) for the combinations of these 3 life context factors together – i.e. those who exercised frequently, experienced no listed traumas/adversities, and had high income.

**Developing a supervised learning model**

All analyses were carried out using Python (version 3.8) including the scikit-learn, pandas, seaborn and shap libraries. Orange (version 3.32), an open-source Python library with a hierarchically-organized toolbox of data mining components, was used to simplify data manipulation, transformation, visualization, and modeling workflows.
